# Supplementary material for: Beyond Seamless: Unexpected Defective Merging in Single‐Orientation Graphene
Source: Small Sci. 2026 Mar 13;6(3):e202500609. doi: 10.1002/smsc.202500609 (PMC13097498; doi:10.1002/smsc.202500609)
Supplement: Supplementary file 1 — Supplementary Material [file SMSC-6-e202500609-s001.pdf]

Supporting Information

Beyond Seamless: Unexpected Defective Merging in Single-Orientation Graphene

Zhien Wang[1,2,†], Jiangtao Wang[2,,†], Diego Exposito[3], Andrey Krayev[4], Shih-Ming He[2], Xudong Zheng[2], Zachariah Hennighausen[2], Ivan Brihuega[3,5], Se-Young Jeong[6,7,*], Jing Kong[2,*]*

[1]Department of Materials Science and Engineering, Massachusetts Institute of Technology, Cambridge, MA 02139, United States

[2]Department of Electrical Engineering and Computer Science, Massachusetts Institute of Technology, Cambridge, MA 02139, United States

[3]Departamento de Física de la Materia Condensada, Universidad Autónoma de Madrid, E-28049 Madrid, Spain

[4]HORIBA Scientific, HORIBA Instruments Incorporated, 359 Bel Marin Keys Blvd, Suite 18, Novato, CA, 94949, USA

[5]Condensed Matter Physics Center (IFIMAC), Universidad Autónoma de Madrid, E-28049 Madrid, Spain

[6]Department of Optics and Mechatronics, Pusan National University, Busan 46241, South Korea

[7]Department of Physics, Korea Advanced Institute of Science and Technology (KAIST), Daejeon 34141, Republic of Korea.

*Corresponding authors: Jiangtao Wang, Se-Young Jeong, Jing Kong

Email: wangjt@mit.edu, syjeong@pusan.ac.kr, jingkong@mit.edu

Graphene Growth and Characterizations.

To develop an optimal recipe for high-quality graphene synthesis on 700nm single-crystalline Cu(111) thin film, we evaluated growth parameters across different pressure regimes to balance film continuity, grain size and quality. Initial efforts using low-pressure CVD (LPCVD) faced significant challenges due to the rapid evaporation of the copper thin film, and growth process highly sensitive to oxygen; while trace oxygen reduced nucleation density, higher concentrations caused particle formation via reaction with silicon. By installing filters to remove oxygen and water from the precursors, we achieved a high nucleation density that enabled the rapid formation of a continuous film (flake size 3 µm) within 5 minutes (**Figure S1**a-d), effectively minimizing copper loss due to evaporation. However, this method suffered from batch-to-batch variability. Raman analysis on transferred graphene revealed p-doping, and bilayers are difficult to remove (Figure S1e-f). Consequently, to establish a more reproducible and stable recipe, we transitioned to investigating ambient pressure (AP) CVD. This approach significantly mitigated copper evaporation and, despite requiring a longer growth time of 120 minutes, yielded superior crystallinity with grain sizes reaching up to 100 µm. Characterization of the transferred APCVD graphene confirmed its high quality, exhibiting a 2D/G ratio around 2.6, a negligible D peak, and a nearly doping-free scatter plot (**Figure S2**).

It is also worth noting that while we compared low-pressure and ambient-pressure regimes to optimize overall film quality, We did not perform a systematic study to determine how specific growth variables influence the ratio of seamless stitching to overlapped junctions. Although literature

suggests that factors such as high hydrogen partial pressure may increase the probability of forming overlapped junctions [27], our use of 700 nm Cu(111) thin films imposed a narrow growth window; deviating from these optimized conditions to tune the stitching modes would have risked substrate evaporation or bilayer formation.

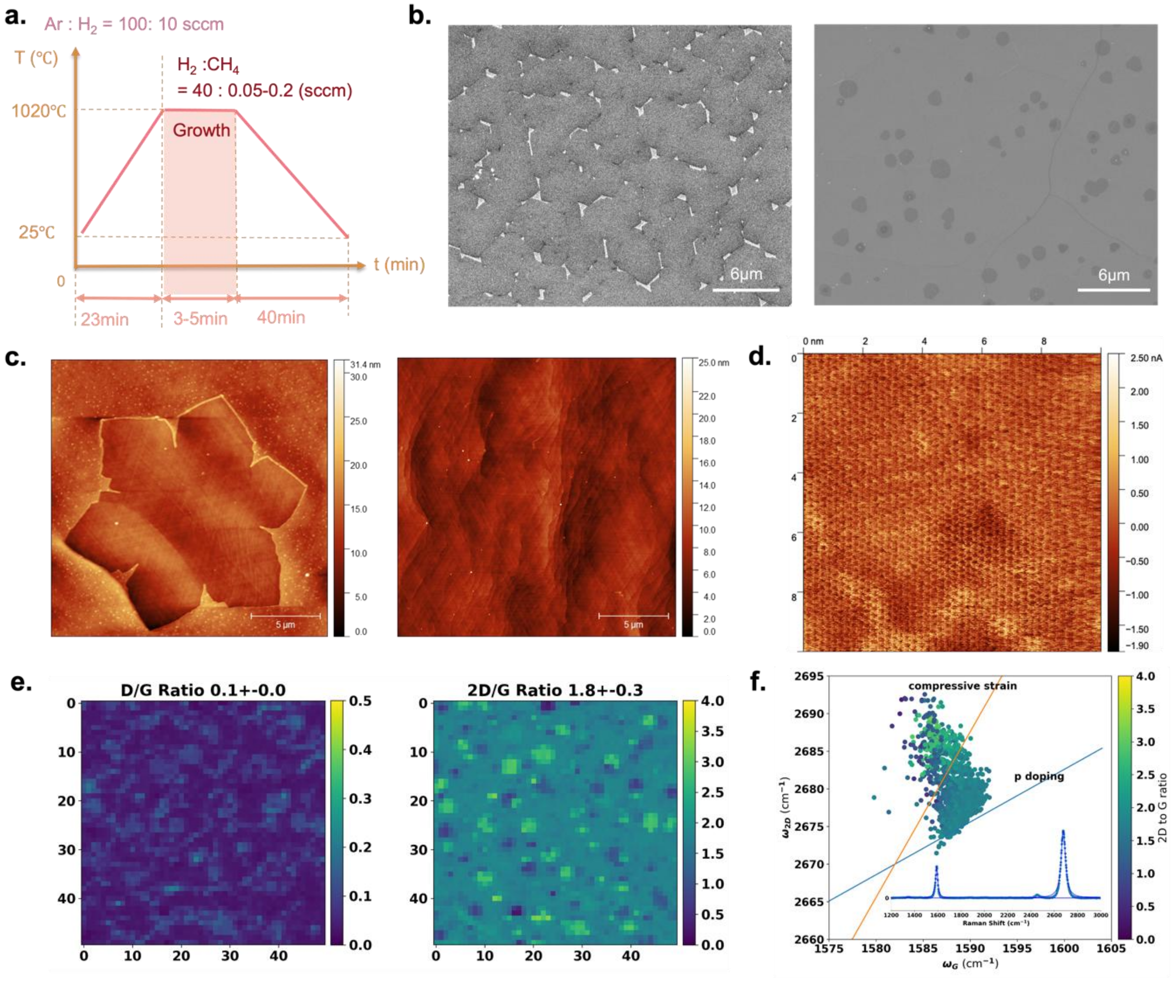

Figure S1. (a) Schematic of the low-pressure (LP) CVD recipe used for growing graphene on a 700 nm Cu(111) film. **(b)** Scanning Electron Microscope (SEM) image of the graphene immediately after growth on the copper substrate. **(c)** Atomic Force Microscopy (AFM) scans of the as-grown graphene. Note: The copper film was oxidized post-growth to enhance the visibility of graphene flakes during optical microscopy. **(d)** Conductive AFM (cAFM) image showing the graphene structure at atomic resolution. **(e)**

Raman spectroscopy maps illustrating the D/G and 2D/G intensity ratios for a 50 µm × 50 µm area of graphene transferred onto a SiO_2 substrate. **(f)** Scatter plot correlating Raman G-band and 2D-band frequencies, based on 2,500 data points collected from the transferred graphene sample.

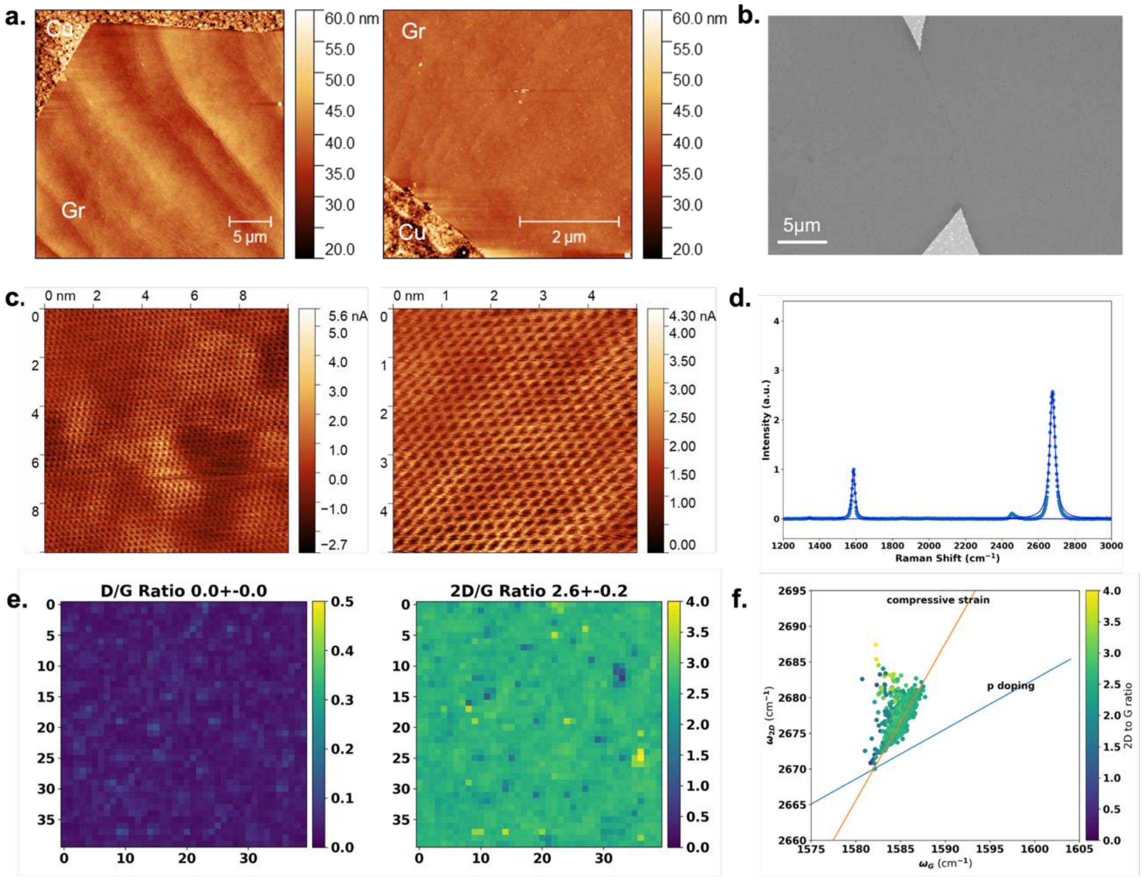

Figure S2. (a) AFM images of as-grown graphene on Cu(111) film. Cu(111) film is oxidized after growth to locate the graphene flake under optical microscopy (b) SEM image of as-grown graphene on Cu(111) film (c) Atomic resolution cAFM image of graphene. (d) A typical Raman spectrum obtained from the sample. (e) Raman mapping of D/G intensity ratio and 2D/G ratio for 40μm×40μm graphene transferred on SiO_2 (f) Raman scattering plot of 1600 points, plotted along with the compressive strain and p doping line.

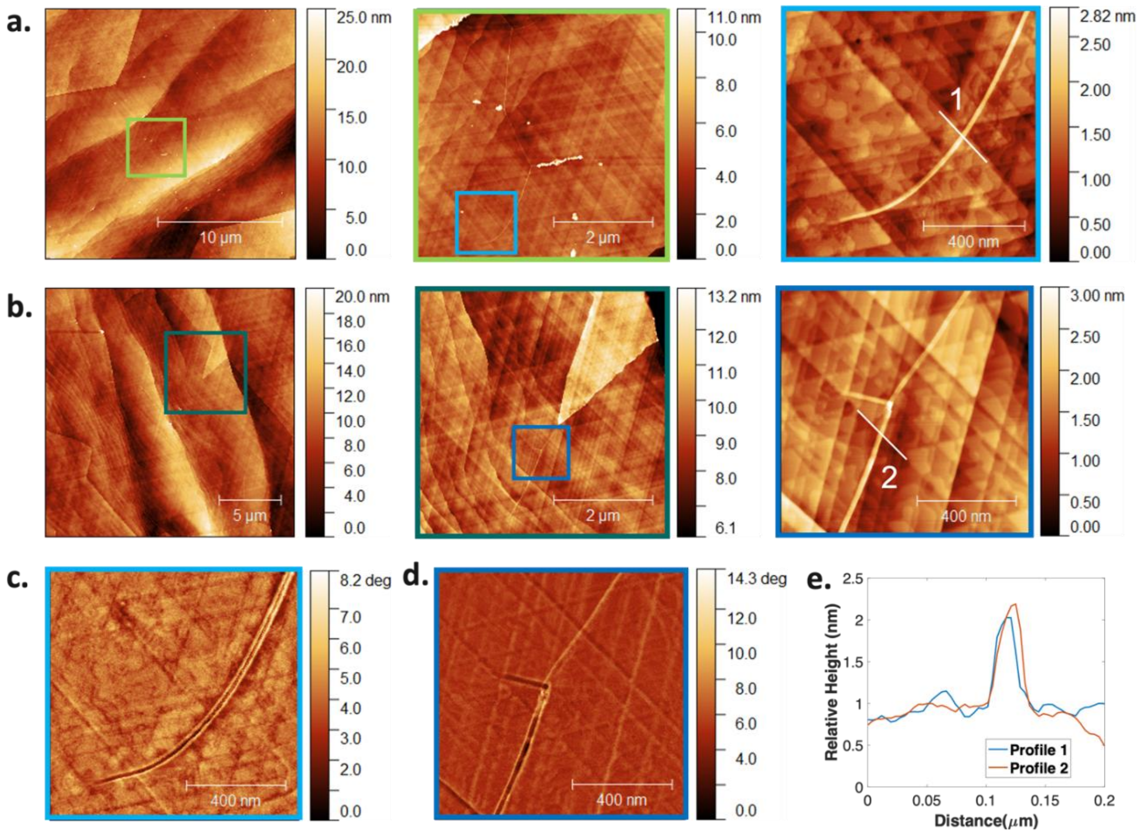

Figure S3. AFM profiles of wrinkles on graphene. (a) AFM topography profile of a wrinkle at the merging region of two flakes (indicated by the light green box). (b) AFM topography profile of a wrinkle on another merging region (indicated by the dark green box). (c) AFM phase profile of the wrinkle in (a). (d) AFM phase profile of the wrinkle in (b). (e) Height measurement of wrinkles in (a) and (b). Both are > 1nm.

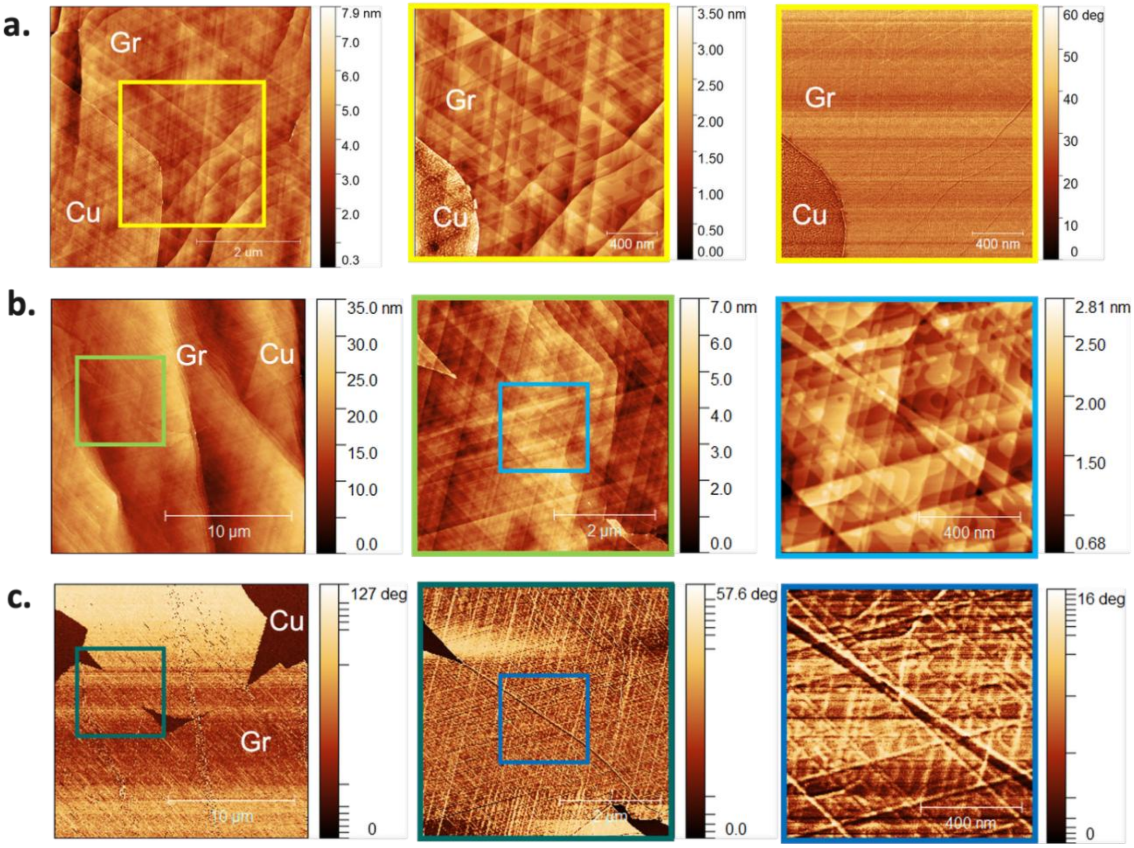

Figure S4. Areas with & without the merging lines under AFM. (a) Area 1: merging graphene flakes with no line, (b) & (c) Area 2: topography and phase profile of merging graphene flakes with the merging line

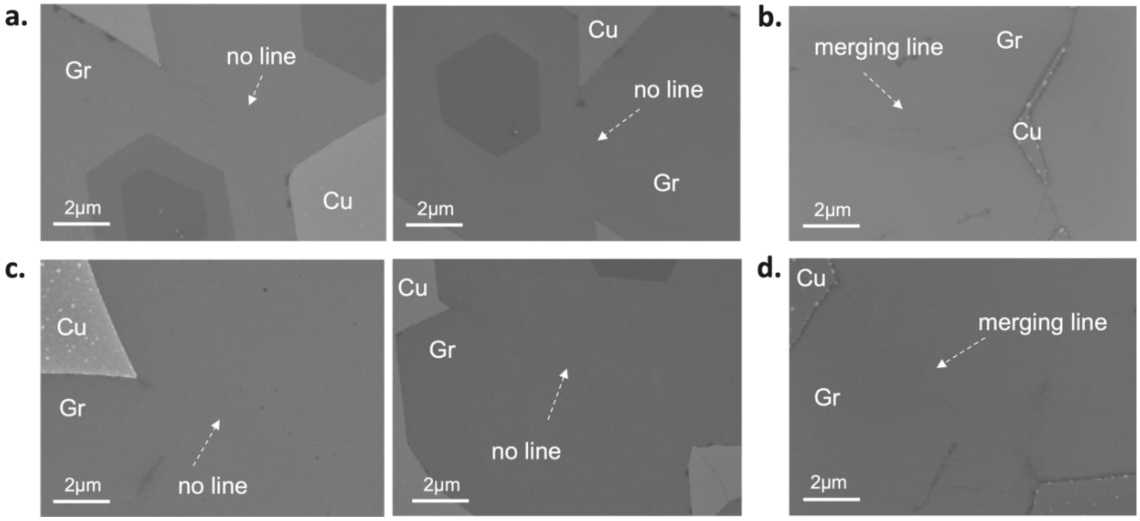

Figure S5. Six areas with & without a merging line at the same magnification under SEM. (a)&(c) Four merging areas show no merging line under SEM. (b)&(d) Merging line observed for two graphene flakes stitching together.

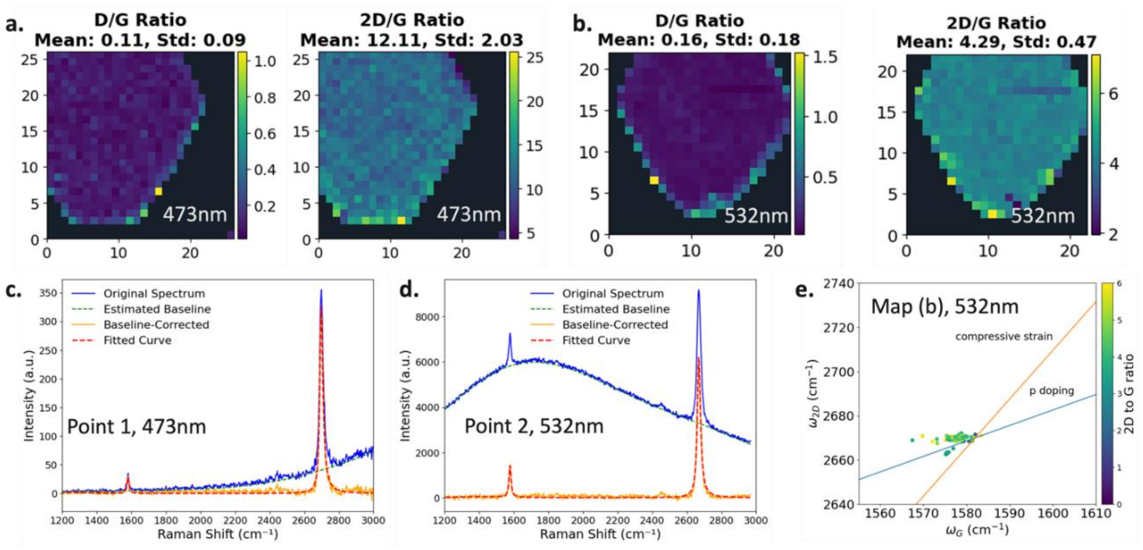

Figure S6. Raman mapping of two graphene flakes using 532nm laser and 473nm laser. (a) Mapping of D/G and 2D/G peaks intensity ratio by 473nm laser. (b) Mapping of D/G and 2D/G peaks intensity ratio by 533nm laser. (c) A Raman spectrum of the graphene flake in (a) by 473nm laser, with fittings of G and 2D peaks. (d) A Raman spectrum of the graphene flake in (b) by 532nm laser, with fittings of G and 2D peaks. (e) Raman scattered plot of the mapping data in graphene flake (b). Most points are close to the intersection between the compressive strain line and p doping line, which indicates no compressive strain or p doping. This shows the graphene is decoupled from the copper substrate after wet oxidation.

Raman maps of position and intensity of G, 2D, and Cu_2O peaks in position 1-3 after wet oxidation

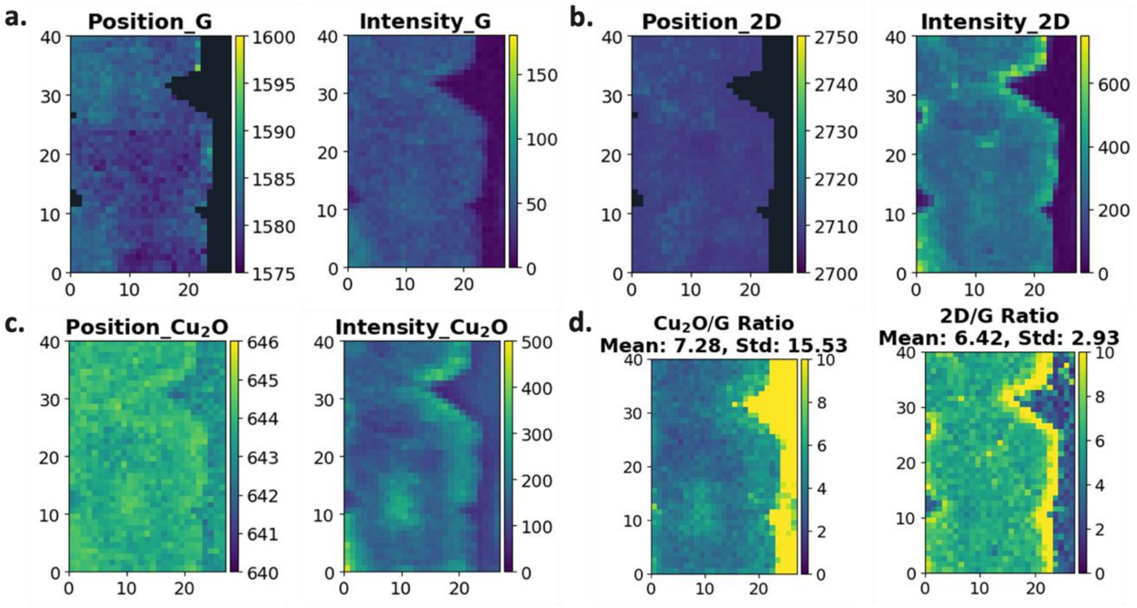

Figure S7. Raman mapping of Position 1. Position and intensity maps of (a) G peak. (b) 2D peak (c) Cu_2O peak (d) Cu_2O/G and 2D/G peak intensity ratio.

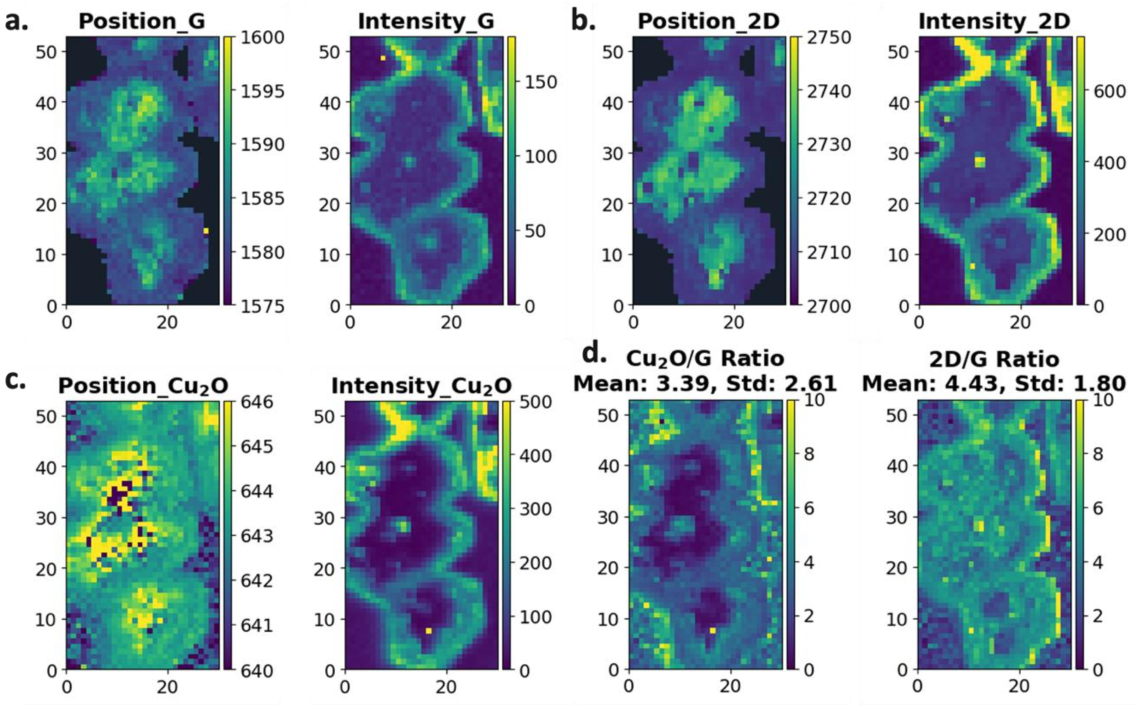

Figure S8. Raman mapping of Position 2. Position and intensity maps of (a) G peak. (b) 2D peak (c) Cu_2O peak (d) Cu_2O/G and 2D/G peak intensity ratio. For this sample, the center region of the graphene flakes is not oxidized yet, accounting for lack of Cu_2O signal (refer to Figure 2 in the main text for detailed explanation).

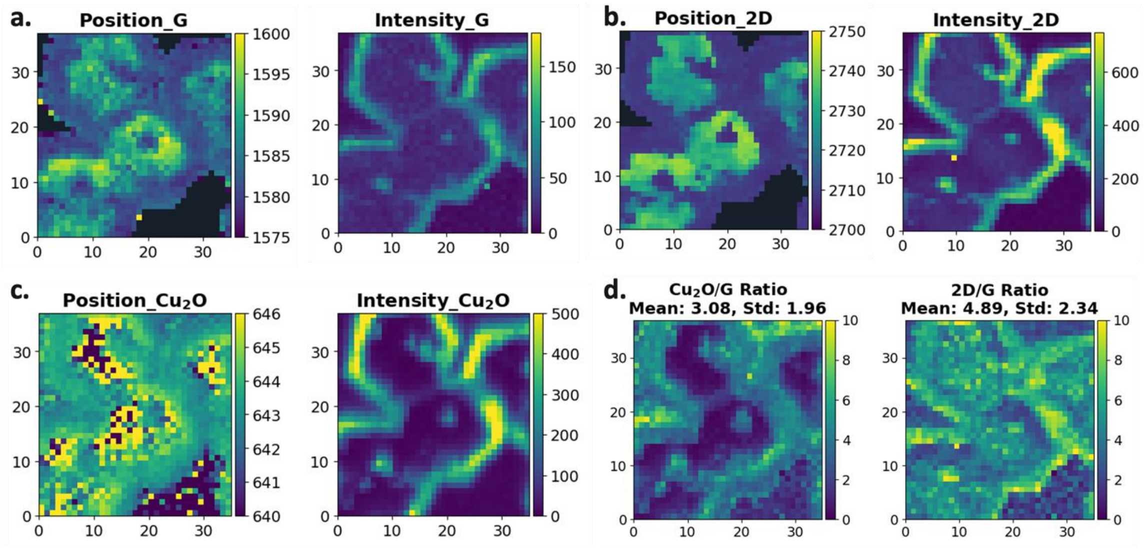

Figure S9. Raman mapping of Position 3. Position and intensity maps of (a) G peak. (b) 2D peak (c) Cu2O peak (d) Cu2O/G and 2D/G peak intensity ratio. The center region of graphene flakes is not oxidized in this process, therefore no Cu2O Raman signal (refer to Figure 2 in the main text for detailed explanation).

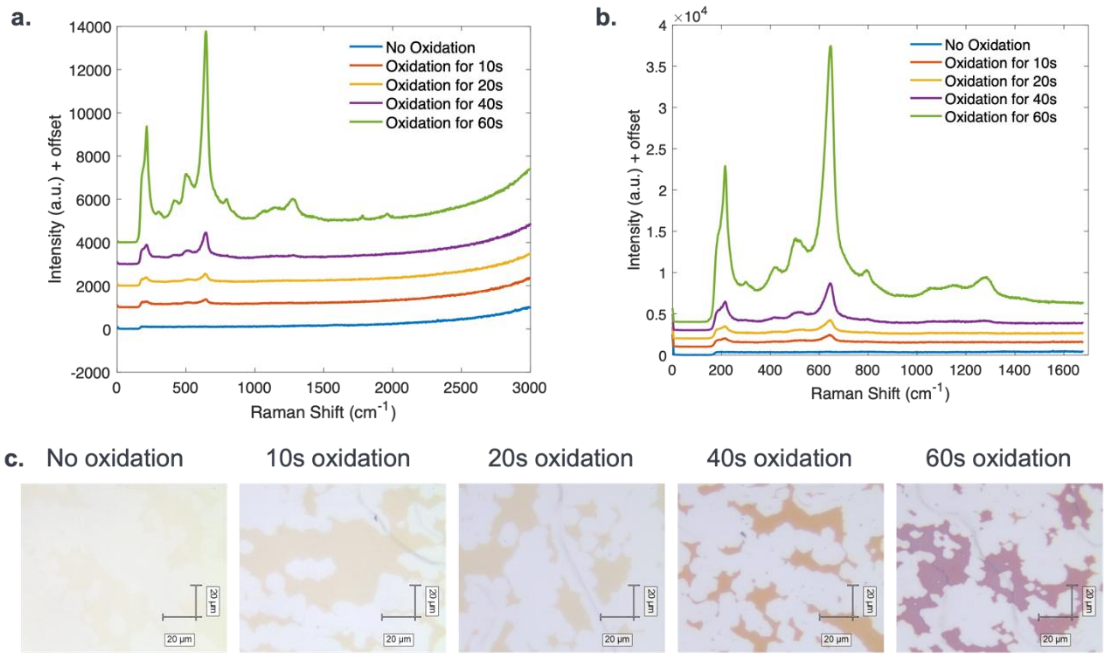

Figure S10. Raman spectrum of the Cu(111) film under different oxidation time (a) Raman spectrum of Cu(111) film (not on graphene) under different oxidation time (473nm laser, 1200 grating). (b) Raman spectrum of Cu(111) under different oxidation time (473nm laser, 2400 grating). (c) Optical microscopic image of graphene flakes on Cu(111) film under different oxidation time

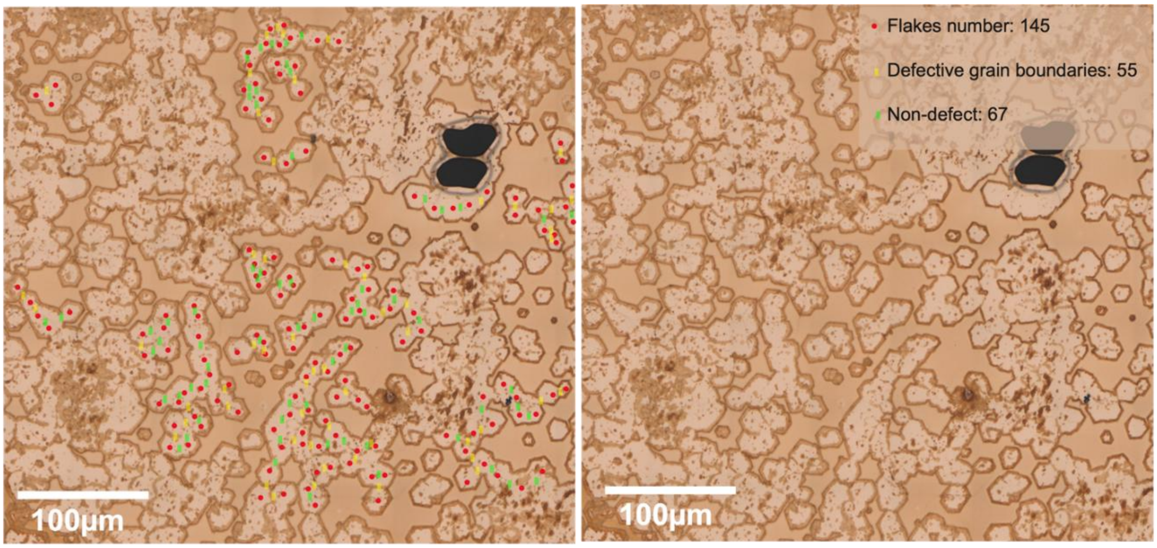

Figure S11. Estimation based on an optical microscopic image of defective stitches vs. seamless stitches from 145 merging graphene flakes, after wet oxidation. Left: Optical microscopic image with annotations. Right: original optical microscopic image.

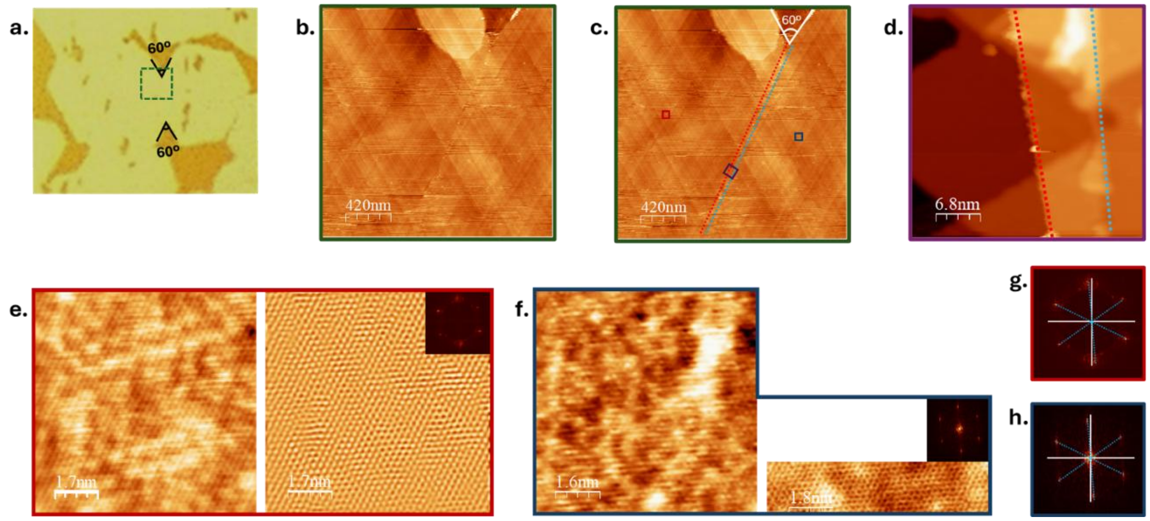

Figure S12. STM imaging of the merging line from two aligned graphene flakes.

(a) Optical microscopy image of two merging graphene flakes. The angle between the edges of the graphene flakes, both in optical microscope (a) and STM images (c), is 60°, indicating that both merging flakes are of aligned orientation. (b) STM image of the region outlined by a green square in (a). (c) Same image as (b), with red and blue dashed lines highlighting the overlapped area. The purple square indicates the position of Figure4b. (d) STM image of the purple region at (c), note that it is rotated. The overlapping region (where the two graphene flakes meet) is confined between the dashed lines shown in the image. The red dashed line marks the edge of the upper graphene layer, corresponding to the flake on the right-hand side of the merging line. The blue dashed line indicates the edge of the bottom graphene layer, belonging to the flake on the left-hand side of the merging line (e) Atomic-resolution image on the left graphene flake at the position outlined with a red square in (c). Left, raw data. Right, FFT low pass filtered image highlighting the atomic structure of graphene, the inset being the extracted FFT. (f) Atomic- resolution images on the right graphene flake at the position outlined with a blue square in (c). Inset shows the extracted FFT. (g),(h) Comparison between the extracted FFTs for atomic-resolution images at both sides of the overlapped region. They show the same atomic orientation, further confirming that the merging flakes are aligned.

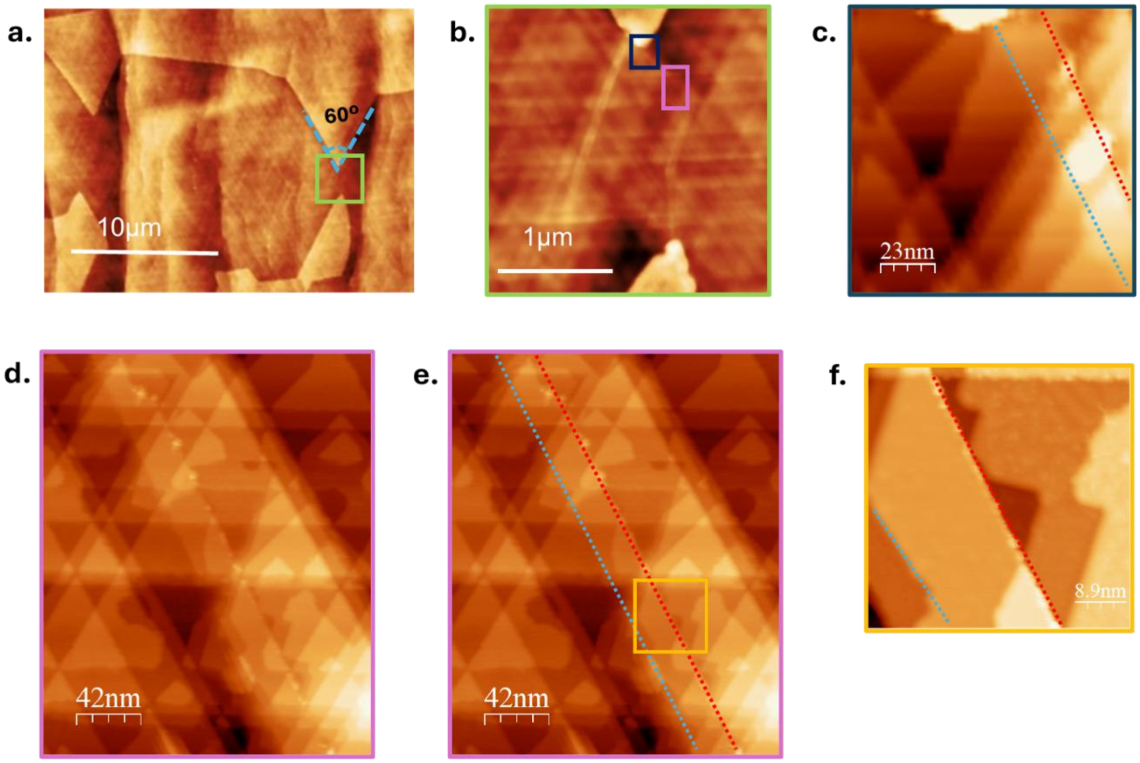

Figure S13. STM imaging of merging lines. (a) AFM image of two merging graphene flakes with aligned orientation, as indicated by the 60º angle between the edges of both graphene grains, outlined at the top of the merging region. (b) AFM image of the region outlined by the green square in (a). The two rectangles, pink and blue, indicate the two areas of the merging lines characterized by STM, Figure 4e-g and Figure 4h-k respectively. (c) STM image of the overlapping area at the blue square in panel (b), corresponding to Fig 4h. The overlapping region is confined between the dashed lines shown in the image. The red dashed line marks the edge of the upper graphene layer, corresponding to the flake on the left-hand side of the merging line. The blue dashed line indicates the edge of the bottom graphene layer, belonging to the flake on the right-hand side of the merging line. (d) STM image of the region outlined by a pink rectangle in (a). (e) Same image as (d), with red and blue dashed lines highlighting the overlapped area. The yellow square indicates the position of Figure4e. (f) STM image at the yellow square position in (e). The overlapping region is confined between the red and blue dashed lines outlining its borders.

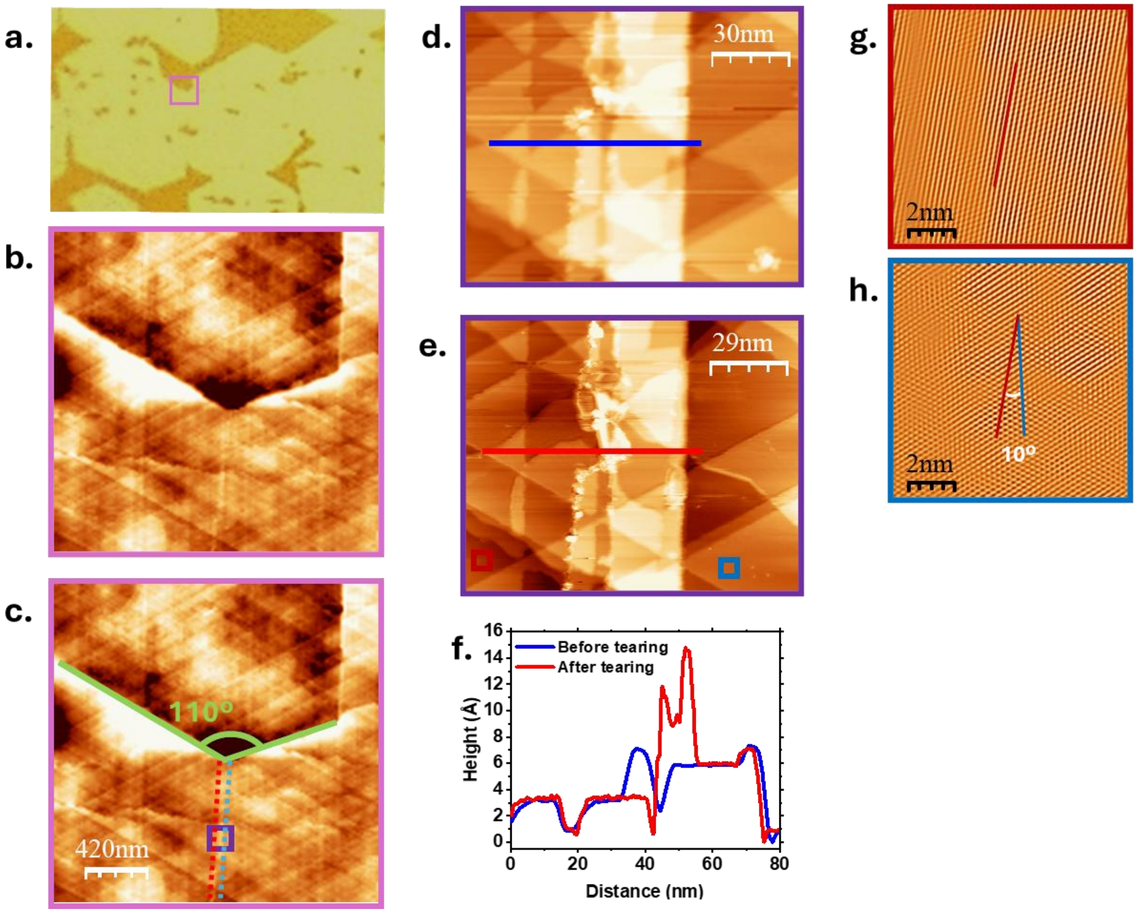

Figure S14. STM imaging of the merging line of two graphene flakes with 10° mismatch in orientation. (a) Optical microscope image of two misaligned merging graphene flakes. (b) STM image of the region outlined by a pink square in (a). (c) Same image as (b). The angle between both merging graphene flakes form 110º, indicating a 10-degree misalignment. Red and blue dashed lines highlight the overlapping region (d) STM imaging of the area indicated as a purple box in (b), an overlapping region of around 30-50nm wide is clearly seen. (e) Tearing happens during STM scanning over a highly defective region of the upper edge of the merging line. (f) Height profile across the merging line measured before (d) and after (e) the tearing. The step height measurements across the merging line support the structure of an overlapped junction. (g), (h) FFT Filtered STM images highlighting graphene's atomic structure from both merging flakes, measured at the positions indicated in (e) by the red and blue squares. The 10° misalignment between the atomic lattices of both graphene flakes matches the 10° misalignment angle observed between their edges in (c).

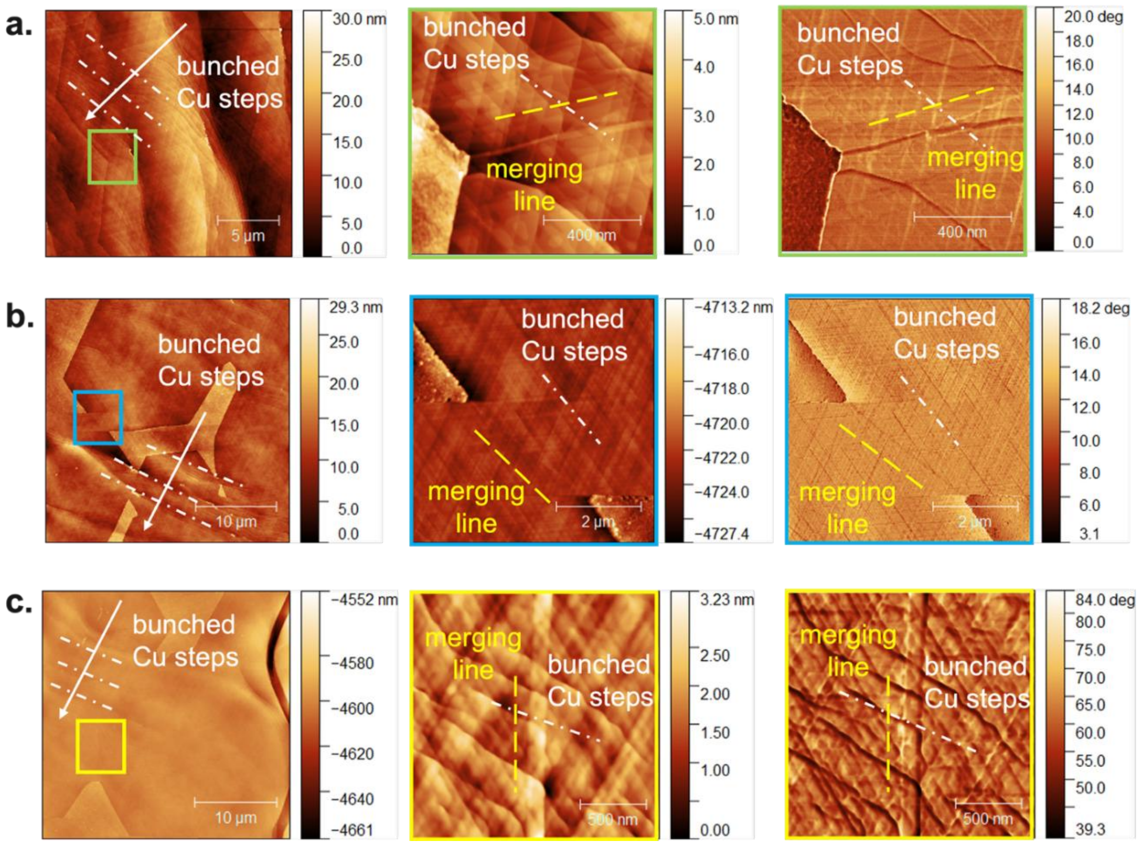

Figure S15. Merging lines in different angles with regards to the direction of bunched Cu steps. (a)&(c) AFM image of two merging graphene flakes, where the merging line is in a small angle with the direction of bunched Cu steps. (b) AFM image of two merging graphene flakes, where the merging line is in nearly 90 degrees with the direction of bunched Cu steps.

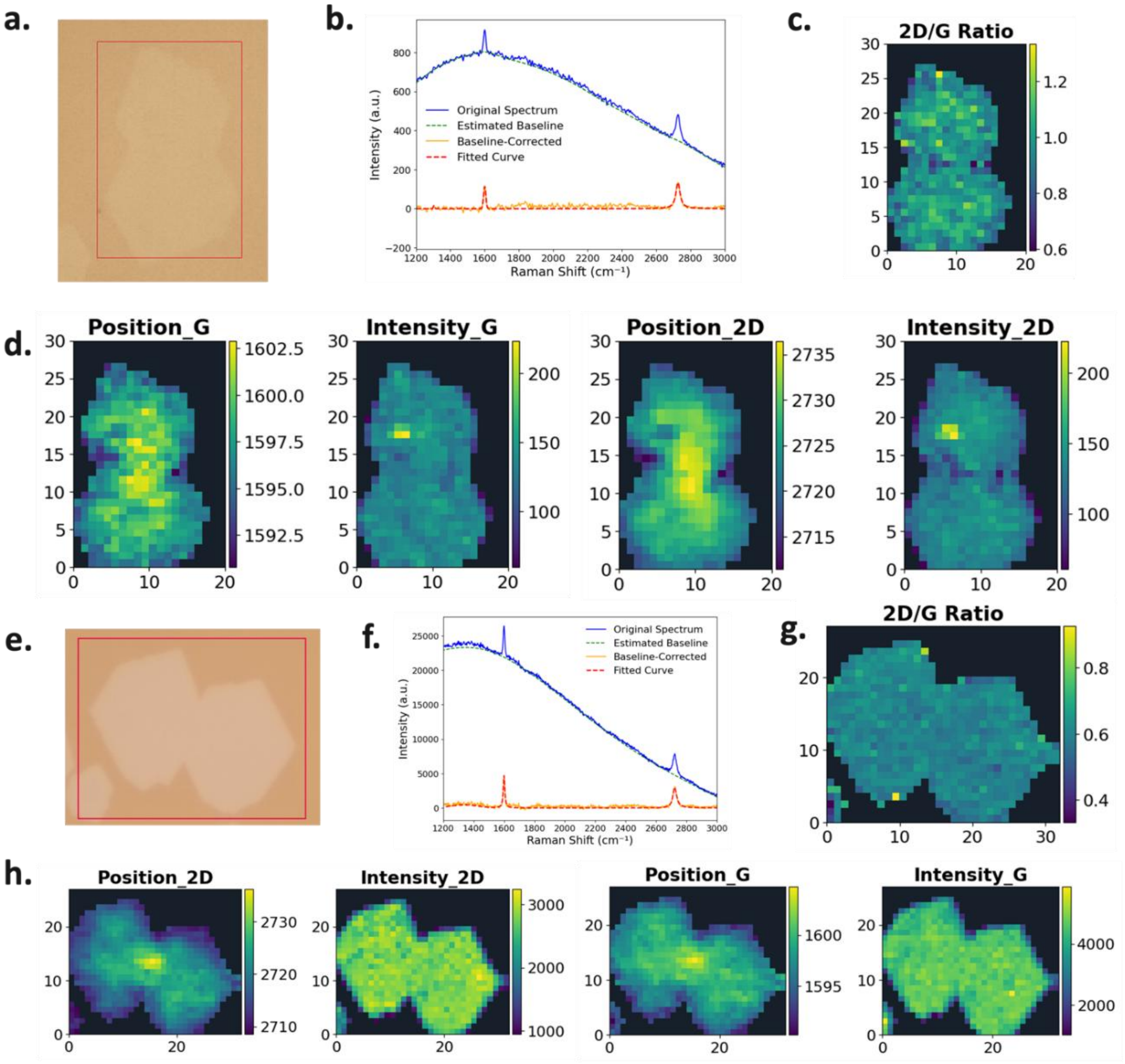

Figure S16. Higher compressive strain at the merging area of two aligned graphene flakes. (a) Area 1 of two merging graphene flakes. (b) Raman spectrum of one spot in area 1 with fitting. (c) Raman map of 2D/G peak intensity ratio on area 1. (d) Raman map of G and 2D peak position and intensity of area 1. (e) Area 2 of two merging graphene flakes. (f) Raman spectrum of one spot in area 2 with fitting. (g) Raman map of 2D/G peak intensity ratio on area 2. (h) Raman map of G and 2D peak position and intensity of area 2. Red-shift of both G and 2D peak positions at the merging area, indicating higher compressive strain at the merging area. Raman map in area 1 is obtained with a lower laser power to preserve the graphene for further characterization, which accounts for the lower intensity in G and 2D peaks.
